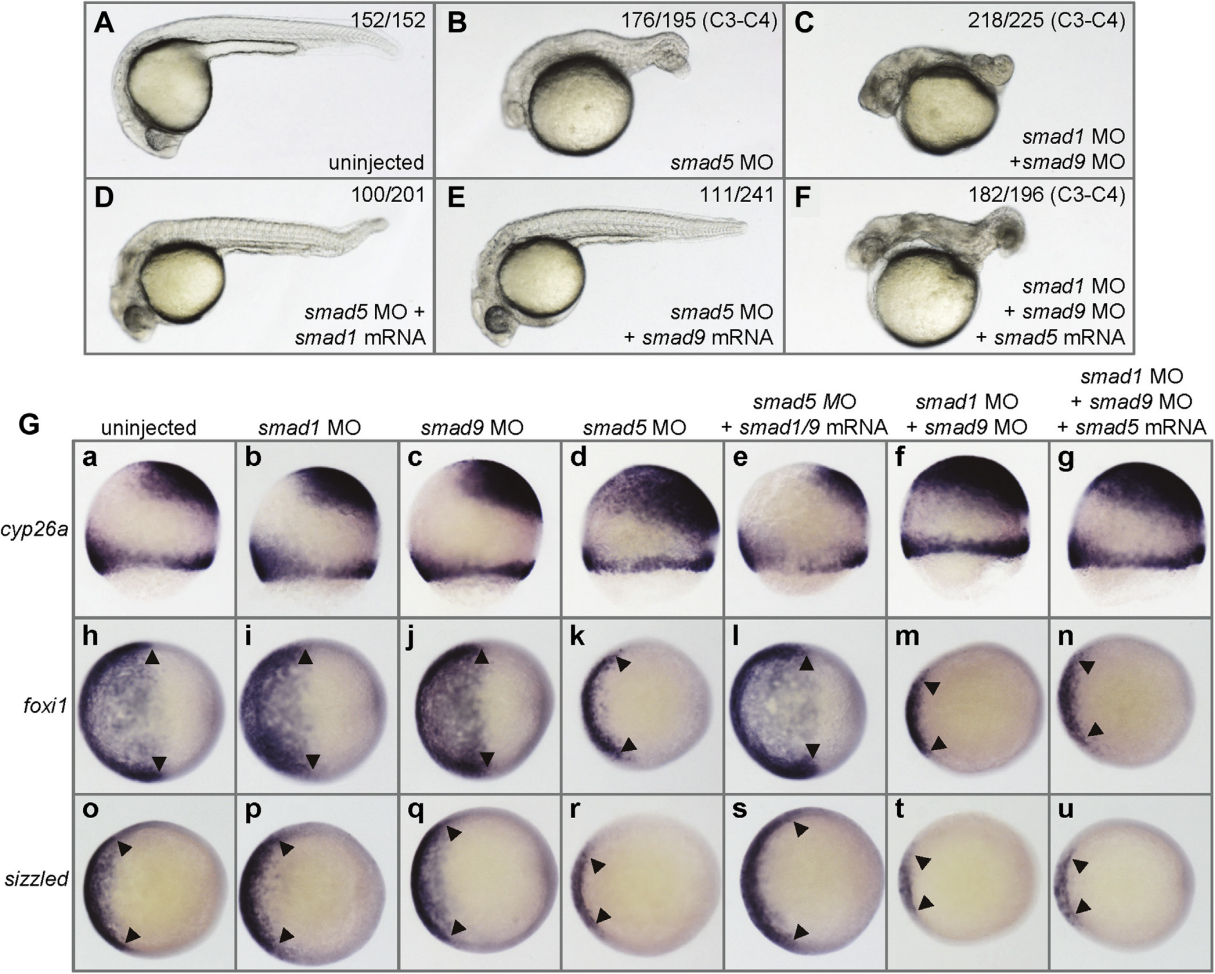# Correction: Transcriptional factors smad1 and smad9 act redundantly to mediate zebrafish ventral specification downstream of smad5

**DOI:** 10.1016/j.jbc.2020.100158

**Published:** 2021-02-13

**Authors:** Chang-Yong Wei, Hou-Peng Wang, Zuo-Yan Zhu, Yong-Hua Sun

VOLUME 289 (2014) PAGES 6604–6618

In Fig. 4*G*, in the *foxi1* panel, the images in Figs. 4*G(i)* and 4*G(l)*, corresponding to ‘*smad1* MO’ and ‘*smad5* MO + *smad1/9* mRNA’ samples respectively, were inadvertently reused during figure preparation. This error has now been corrected using images pertaining to each treatment and sample. This correction does not affect the results or conclusions of the work.

**Figure:**